# Concurrent Infection with Hepatitis C Virus and *Streptococcus pneumoniae*

**DOI:** 10.3201/eid2307.161858

**Published:** 2017-07

**Authors:** Thomas J. Marrie, Gregory J. Tyrrell, Sumit R. Majumdar, Dean T. Eurich

**Affiliations:** Dalhousie University, Halifax, Nova Scotia, Canada (T. J. Marrie);; Provincial Laboratory for Public Health, Edmonton, Alberta, Canada (G.J. Tyrrell);; University of Alberta, Edmonton (G.J. Tyrrell, S.R. Majumdar, D.T. Eurich)

**Keywords:** hepatitis C, hepatitis C virus, HCV, viruses, Streptococcus pneumoniae, bacteria, streptococci, invasive pneumococcal disease, IPD, respiratory infections, prevalence, correlates, outcomes, concurrent infection, co-infection, mortality rates, complications, serotypes, Alberta, Canada

## Abstract

Little is known about concurrent infection with hepatitis C virus (HCV) and *Streptococcus pneumoniae*, which causes invasive pneumococcal disease (IPD). We hypothesized that co-infection with HCV and *S. pneumoniae* would increase risk for death and complications. We captured sociodemographic and serologic data for adults with IPD in a population-based cohort study in northern Alberta, Canada, during 2000–2014. IPD patients infected with HCV were compared with IPD patients not infected with HCV for risk of in-hospital deaths and complications by using multivariable logistic regression. A total of 355 of 3,251 patients with IPD were co-infected with HCV. The in-hospital mortality rate was higher for IPD patients infected with HCV. Prevalence of most IPD-related complications (e.g., cellulitis, acute kidney injury, mechanical ventilation) was also higher in HCV-infected patients. Infection with HCV is common in patients with IPD, and HCV is independently associated with an increased risk for serious illness and death.

Hepatitis C virus (HCV) is a flavivirus that can cause a chronic hepatic infection in 75%–85% of persons initially infected (*1*). This virus is present worldwide, infects 130–175 million persons, and causes sequelae that include cirrhosis, end-stage liver disease, and hepatocellular cancer (*1*). In some patients infected with HCV, bacterial infections are more common and more severe than in patients not infected with HCV (*2*–*6*). Co-infection with HIV is also common among HCV-infected patients and can lead to higher mortality rates from sepsis than infection with either virus alone (*4*). Bacteremia has also been shown to be higher in hemodialysis patients infected with HCV (38.3%) than those not infected (21.8%), and mortality rates are higher for bacteremic patients infected with HCV than in those not infected (*3*).

*Streptococcus pneumoniae* is a gram-positive bacterium found in the nasopharynx and is a common cause of bacteremia. This microorganism can cause a variety of diseases ranging from noninvasive disease, such as pneumonia without bacteremia, to invasive pneumococcal disease (IPD), such as pneumonia with bacteremia, primarily in young or elderly persons (*5*,*6*). The virulence of this bacterium is driven by its wide assortment (currently 97) of polysaccharide capsule types (serotypes) (*7*).

To improve understanding of IPD and the effects of the use of pneumococcal protein conjugate vaccines introduced into Alberta, Canada, in 2002 and 2010, we began a 15-year longitudinal study of IPD in 2000. We took advantage of this prospective cohort to examine the prevalence, correlates, and outcomes of IPD patients co-infected with HCV. We hypothesized that IPD patients co-infected with HCV would have higher mortality rates and more complications than IPD patients not infected with HCV.

## Materials and Methods

### Definitions

In Canada, the national case definition for IPD is isolation of *S. pneumoniae* from a normally sterile site, such as blood, cerebrospinal fluid, pleural fluid, biopsy tissue, joint aspiration, pericardial fluid, or peritoneal fluid (*8*). IPD is a provincially reportable disease in Alberta. Therefore, all invasive pneumococcal isolates are submitted to the Provincial Laboratory for Public Health (Edmonton, Alberta, Canada) for further characterization. Access to these isolates enabled us to prospectively identify all cases of IPD in northern Alberta. However, the study was limited to persons >17 years of age. HCV infection was defined as a positive antibody test result for this virus documented in the chart of a patient as part of usual care (i.e., we did not specifically test each patient for HCV for purposes of this study).

### Clinical Data Collection

Research nurses collected sociodemographic, clinical, functional, and laboratory data from the medical record of each patient by using a standardized case report form (Tables 1, 2). These nurses received training on data collection before the start of the study. In addition to the case report form, standard operating procedures documents, definitions, drug classification, and underlying illness categorization were part of working documents. With respect to concurrent illnesses, if the attending physician recorded such an illness, it was accepted. Complete data were available for sociodemographic and functional status. If there was no record of a concurrent illness, it was assumed that the illness was not present. Additional details of the methods used in the study have been reported (*9*). This study was approved by the institutional research review committees of the Alberta Health Regions and the ethics review board of the University of Alberta.

**Table 1:** Characteristics of 3,251 IPD patients >17 years of age by HCV status, Alberta, Canada, 2000–2014*

Characteristic	HCV-positive	HCV-negative	p value
Total no.	355 (10.9)	2,896 (89.1)	
Sex			
M	203 (57.2)	1,635 (56.5)	0.810
F	152 (42.8)	1,261 (43.5)	NA
Mean (SD) age, y	45.2 (9)	55.7 (18.4)	<0.001
Aboriginal	95 (26.8)	355 (11.6)	<0.001
Residence at time of admission			
Home	220 (62.0)	2,408 (83.3)	NA
Homeless	88 (24.8)	148 (5.0)	<0.001
Functional status			
Walking independently	264 (74.4)	2,107 (72.8)	0.520
Concurrent condition			
HIV infection	81 (22.8)	55 (1.9)	<0.001
Cancer in past 5 y	19 (5.4)	385 (13.4)	<0.001
Diabetes	17 (4.8)	179 (6.1)	0.300
Coronary heart disease	16 (4.5)	437 (15.1)	<0.001
Chronic obstructive pulmonary disease	32 (9.0)	532 (18.4)	<0.001
Cirrhosis	86 (24.2)	91 (3.1)	<0.001
>2 other chronic conditions	160 (45.1)	1,354 (46.8)	0.550
Lifestyle factors			
Current smoker	245 (69)	1,222 (42.2)	<0.001
Illicit drug use	236 (66.5)	323 (11.1)	<0.001
Alcoholism	217 (61.1)	568 (19.6)	<0.001

*Values are no. (%) unless otherwise indicated. HCV, hepatitis C virus; IPD, invasive pneumococcal disease; NA, not applicable.

**Table 2:** Manifestations and complications of IPD patients stratified by HCV infection, Alberta, Canada, 2000–2014*

Characteristic	HCV-positive, no. (%)	HCV-negative, no. (%)	Fully adjusted OR (95% CI)	p value
In-hospital death	57 (16.1)	429 (14.8)	1.71 (1.15–2.54)	0.008
Pneumonia	304 (85.6)	2,388 (82.5)	0.82 (0.57–1.20)	0.310
Cellulitis	24 (6.8)	59 (2)	3.40 (1.81–6.39)	<0.001
Septic arthritis	5 (1.4)	46 (1.6)	1.00 (0.35–2.86)	1.000
Endocarditis	4 (1.1)	24 (0.8)	0.93 (0.27–3.21)	0.910
Empyema	30 (8.4)	188 (6.5)	1.11 (0.69–1.80)	0.660
Renal failure requiring dialysis	26 (7.3)	94 (3.2)	2.14 (1.23–3.72)	0.007
Liver failure	21 (5.9)	49 (1.7)	2.70 (1.41–5.18)	0.003
Mechanical ventilation	110 (31)	575 (19.9)	1.44 (1.07–1.94)	0.017
Peritonitis	16 (4.5)	29 (1.0)	3.79 (1.75–8.17)	0.001
Meningitis	6 (1.7)	154 (5.3)	0.38 (0.16–0.92)	0.001

*HCV, hepatitis C virus; IPD, invasive pneumococcal disease.

### Identification and Serotyping of *S. pneumoniae* Isolates

*S. pneumoniae* isolates were received at the Provincial Laboratory for Public Health from acute diagnostic laboratories in Alberta as per requirements of provincial notifiable disease regulations. *S. pneumoniae* isolates were confirmed as *S. pneumoniae* on the basis of characteristic morphology and optochin susceptibility before serotyping (*10*). All pneumococcal isolates that showed a positive Quellung reaction with commercial type-specific antisera (Statens Serum Institute, Copenhagen, Denmark) were assigned a serotype designation (*11*). Strains that were susceptible to optochin but that could not be serotyped by the Quellung assay were assayed by using the AccuProbe *S. pneumoniae* Culture Identification Test (Gen-Probe, San Diego, CA, USA) to confirm species identification.

### Exposure and Outcomes

Our independent exposure of interest was a dichotomous variable representing the presence or absence of concurrent HCV infection. Our primary outcome was in-hospital deaths as recorded in patient records. Secondary outcomes included prespecified complications of IPD, defined as the presence of >1 of the following features: cellulitis, osteomyelitis, septic arthritis, endocarditis, empyema, meningitis, acute kidney injury requiring dialysis, liver failure, peritonitis, and need for mechanical ventilation.

### Statistical Analysis

Descriptive data are shown as mean (SD) for continuous variables and number (%) for categorical variables. Differences were evaluated by using the χ^2^ test for categorical variables and the *t*-test for continuous variables. Pneumococcal serotypes according to HCV status were summarized descriptively.

For our main outcome, we completed a series of multivariate logistic regression analyses to evaluate the association between HCV and in-hospital deaths after accounting for increasing degrees of clinical and prognostic data known to be associated with HCV infection or IPD-related outcomes in an effort to examine risk factor stacking. First, we calculated unadjusted estimates of death for HCV-positive patients compared with estimates for HCV-negative patients (reference group). Second, we adjusted for sociodemographic information (i.e., age, sex, Aboriginal status, and residence at time of admission). Third, we adjusted for the following concurrent conditions: solid organ cancer, HIV, diabetes, coronary heart disease, and chronic obstructive pulmonary disease. In addition, we included a marker for the presence of >2 other chronic conditions as a measure of additional illnesses as reported (*12*,*13*). Fourth, we included functional status in our model (independently walking versus not walking before hospital admission) because this factor has better prognostic value than age and disease severity and acts as a proxy for frailty (*14*). Fifth, we included several health behaviors associated with increased risk for HCV infection, including current smoking status, illicit drug use, and alcoholism.

In addition to our primary analysis, we conducted analyses for patients with a history of cirrhosis and for those infected with HIV. Infection with HIV is associated with cirrhosis, and both factors are associated with increased risk for IPD. 

We report adjusted odds ratios (ORs) from logistic regression models with their respective 95% CIs and associated p values. All analyses were conducted by using Stata version 14 (StataCorp LLC, College Station, TX, USA).

## Results

During the 15 years of the study, 3,251 adults >17 years of age were identified as having cases of IPD. Of these adults, 355 (11%) had HCV infections. We obtained sociodemographic characteristics of the 2 population groups, those infected with HCV and those not infected (Table 1). The population with HCV infections had a mean age that was 10 years younger than that for persons without HCV infections (p<0.001). Persons with HCV infections were significantly (all p<0.001) more likely to be Aboriginal, homeless, have concomitant HIV infection and cirrhosis of the liver, have alcoholism, and be current smokers and illicit drug users (Table 1).

### Inpatient Mortality Rates and Complications

Overall, 486 (15%) patients died in a hospital: higher mortality rates were observed for those with concurrent HCV infections (57, 16%) versus no HCV infections (429, 15%). This absolute increased risk for death of 1% was statistically significant in models sequentially adjusted for sociodemographic variables, concurrent conditions, and lifestyle factors (fully adjusted OR [aOR] 1.71, 95% CI 1.15–2.54; p<0.001) (Table 2; Figure). For sequential models, age had the largest effect on the association between HCV and death (OR 1.10 for unadjusted analyses, OR 1.93 when age was added to model). The risk for death by infection with HCV was similar between age groups (persons <65 years of age, OR 1.76 and persons >65 years of age, OR 1.45; p for interaction = 0.86).

**Figure:**
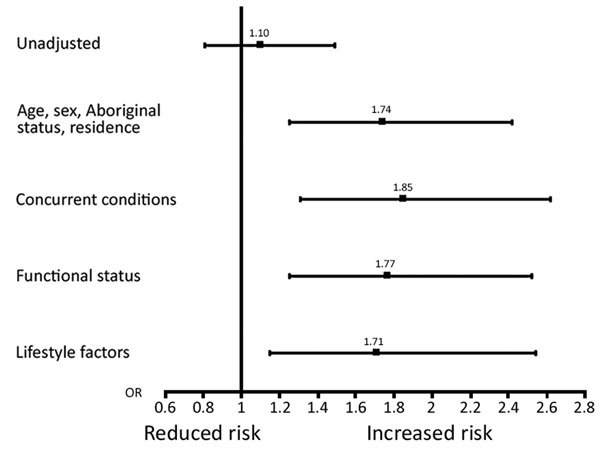
Sequential adjustment for in-hospital deaths, by hepatitis C virus status, Alberta, Canada, 2000–2014. Squares indicate ORs (values shown), and error bars indicate 95% CIs. OR, odds ratio.

Other variables had minor influence on the point estimate for HCV by sequential analyses. Additional variables independently associated with death in a hospital in the final fully adjusted model included increasing age, history of cancer, functional status, smoking status, and alcoholism (p<0.05). Furthermore, patients infected with HCV alone (aOR 1.60) and those infected with HCV who had cirrhosis (aOR 2.85) had an increased risk for death compared with patients who were not infected with HCV and did not have cirrhosis (p for interaction >0.05). Patients infected with HIV alone (aOR 1.71) and those infected with HCV and HIV (aOR 1.46) also had an increased risk for death compared with patients not infected with HCV or HIV (p for interaction >0.05).

In addition to an increased mortality rate, the prevalence of most IPD-related complications, including cellulitis, acute kidney injury, liver failure, peritonitis, and requirement for mechanical ventilation, were higher in HCV-positive patients, but meningitis was significantly lower in HCV-positive patients (p<0.05 for all comparisons) (Table 2).

### Pneumococcal Serotypes

We identified the 10 most common pneumococcal serotypes causing IPD in the 2 study groups (Table 3). There were notable differences between the 2 groups. Serotype 5 caused 19% of infections in HCV-positive patients and 7% of infections in HCV-negative patients. Serotypes 12F, 20, and 9N were approximately twice as common in HCV-positive patients, and 22F was twice as common in HCV-negative patients (Table 3). Overall, 40 serotypes were found in HCV-positive patients and 51 serotypes in HCV-negative patients. Serotypes in 7-valent pneumococcal conjugate vaccine (PCV7), 13-valent pneumococcal conjugate vaccine (PCV13), and 23-valent pneumococcal polysaccharide vaccine caused 18%, 47%, and 71% of infections, respectively, in HCV-positive patients and 24%, 48%, and 70% of infections, respectively, in HCV-negative patients.

**Table 3:** Ten most common *Streptococcus pneumoniae* serotypes causing IPD in HCV-positive and HCV-negative patients, Alberta, Canada, 2000–2014*

Rank	Serotype	HCV-positive, no. (%)	HCV-negative, no. (%)
1	5	67 (18.9)	210 (7.3)
2	4	38 (10.7)	268 (9.3)
3	8	25 (7.0)	236 (8.1)
4	3	22 (6.2)	197 (6.8)
5	12F	20 (5.6)	83 (2.9)
6	20	17 (4.8)	84 (2.9)
7	9N	17 (4.8)	80 (2.8)
8	11A	11 (3.1)	86 (3.0)
9	22F	11 (3.1)	209 (7.2)
10	9V	7 (2.0)	87 (3.0)
Total	NA	235 (66.2)	1, 540 (53.3)

*HCV, hepatitis C virus; IPD, invasive pneumococcal disease; NA, not applicable.

## Discussion

In a prospective cohort of patients with IPD, we found that concurrent infection with HCV was common, as were risk factors for acquisition of HCV, such as Aboriginal status, alcoholism, and illicit drug use. Furthermore, adjusted analyses showed that HCV infection was associated with a 70% increased risk of death. In addition to an increased mortality rate, patients infected with HCV were more likely to have serious complications of IPD other than meningitis.

A recent health technology report from the University of Calgary (Calgary, Alberta, Canada) indicated a prevalence of diagnosed hepatitis C of ≈0.7% in adults in Alberta and a prevalence as high as 3% in Aboriginal persons (*15*). Thus, the overall prevalence rate for hepatitis C of 11% for those with IPD is 15-fold higher than that for the general population and 3.6-fold higher than that for the Aboriginal population. The reasons for this increased prevalence are complicated by known risk factors, such as concurrent tobacco smoking, alcoholism, and homelessness (*16*–*19*). Whether HCV infection only confers a higher risk for IPD requires further study.

We have shown that cirrhosis of the liver is a risk factor for death in patients with IPD (*9*). A rat model of cirrhosis showed multiple impairments of pulmonary defenses against *S. pneumoniae*, including impairment of extrapulmonary killing of the infecting organism before arrival of neutrophils (*20*). Levels of lysozyme and C3 were decreased in bronchoalveolar lavage washings, and neutrophil-mediated killing of the virulent type 3 strain of *S. pneumoniae* was also impaired (*20*).

The prevalence of peritonitis for HCV-positive patients (5%) versus that for HCV-negative patients (1%) in our study was expected, given the likely higher amount of ascites in HCV-positive patients. Empyema complicated the course of 8% of HCV-positive patients compared with 3.4% and 3.3% of HCV-positive patients in previous studies of IPD (*21*–*23*).

A cellulitis prevalence of 7% in HCV-positive patients was ≈3 times the prevalence of 2% for HCV-negative patients. Cellulitis caused by *S. pneumoniae* was reported in 1917 and seems to be an uncommon entity; only 45 cases had been reported as of 2006 (*23*–*26*). Our data indicated that cellulitis complicates IPD much more frequently than previously recognized. In our study, we detected 83 cases of cellulitis. This finding strongly suggests that HCV infection is associated with pneumococcal cellulitis, especially given the aOR of 3.4 we documented (*27*). Moreover, although HCV-positive patients were more likely to be homeless, occurrence of cellulitis was similar irrespective of underlying place of residence and could not explain these findings. A higher rate of liver failure among HCV-positive patients probably indicates that these patients had cirrhosis at time of hospitalization, and IPD triggered liver failure in patients with compromised liver function.

Renal failure was more common in IPD patients infected with HCV. There are several mechanisms whereby renal involvement occurs in HCV-infected patients, including glomerular immune complex disease, direct viral invasion of renal parenchyma, and nephrotoxicity of some drugs used to treat HCV infection (*28*).

A finding that is more difficult to explain is the lower rate of meningitis for HCV-positive than for HCV-negative patients. However, whether meningitis is less frequent in HCV-positive patients remains uncertain. In France, a reduction in pneumococcal meningitis was observed after introduction of PCV13 for children and adults (*29*). Conversely, Moore et al. found that the rate of meningitis for adults increased during the post-PCV13 era when compared with the pre-PCV13 era (*30*).

To explain why meningitis rates were different for HCV-positive and HCV-negative patients in our study cohort, rates of vaccination with PCV13 would also have to be different between HCV-positive and HCV-negative patients. Although possible, we believe that this suggestion is unlikely. An alternative explanation that we believe is more plausible might be that HCV-positive patients are at substantially increased risk for all-cause and IPD-related deaths and that the HCV-positive patients who do not die during hospitalization end up also being at a lower risk for meningitis (e.g., a form of healthy survivor bias) and therefore appear as if this complication is less likely to develop in them. Thus, those patients infected with HCV who died during hospitalization probably also had clinically unrecognized meningitis, rather than having HCV being protective against this complication. Alternatively, it might be possible that patients infected with HCV tended to have lower platelet counts and were therefore less likely to have cerebrospinal fluid collected and a diagnosis of meningitis confirmed.

With respect to serotypes, serotype 5 was most common in the HCV group because of an outbreak of serotype 5 infections that occurred in western Canada and preferentially infected the homeless population (*31*). We also observed an outbreak of infections with serotype 20 among middle-age homeless persons in Edmonton, Alberta, starting in 2012 (G.J. Tyrrell, unpub. data). Serotypes contained in PCV7, PCV13, and 23-valent pneumococcal polysaccharide vaccines had a similar occurrence in HCV-positive and HCV-negative patients, except that HCV-positive patients were less likely to be infected with serotypes in PCV7.

The strengths of this study include the large number of patients with IPD studied over a long period and the richness of the clinical data obtained for each patient. However, our study had limitations. First, we identified HCV-positive patients on the basis of a positive antibody test result. Thus, some patients who were antibody positive but HCV RNA negative would be falsely identified as having active infection with HCV. Second, we were unable to categorize the severity of liver disease. Third, we did not have detailed information regarding the vaccination status of all patients. Fourth, we examined only patients who had a diagnosis of HCV and did not prospectively obtain serologic data for all patients admitted to a hospital with IPD.

In conclusion, we have shown that concurrent HCV infection in patients with IPD is common, and HCV infection in this population portends an increased risk for death and more serious complication of IPD, such as cellulitis and peritonitis (but not meningitis) than for patients not infected with HCV. Because many HCV-infected patients have risk factors that are indications for pneumococcal vaccination, additional studies of IPD in patients infected with HCV are needed to determine whether HCV without other risk factors is an indication for vaccination.
